# Identification of the hub genes associated with prostate cancer tumorigenesis

**DOI:** 10.3389/fonc.2023.1168772

**Published:** 2023-05-12

**Authors:** Honghui Zhu, Qi Lin, Xiaomin Gao, Xixi Huang

**Affiliations:** Department of Urology, The First Affiliated Hospital of Wenzhou Medical University, Wenzhou, China

**Keywords:** differentially expressed genes (DEGs), bioinformatics analysis, hub genes, prostate cancer, biomarkers

## Abstract

**Introduction:**

Prostate cancer (PCa) is one of the most common malignant tumors of the male urogenital system; however, the underlying mechanisms remain largely unclear. This study integrated two cohort profile datasets to elucidate the potential hub genes and mechanisms in PCa.

**Methods and Results:**

Gene expression profiles GSE55945 and GSE6919 were filtered from the Gene Expression Omnibus (GEO) database to obtain 134 differentially expressed genes (DEGs) (14 upregulated and 120 downregulated) in PCa. Gene Ontology and pathway enrichment were performed using the Database for Annotation, Visualization, and Integrated Discovery, showing that these DEGs were mainly involved in biological functions such as cell adhesion, extracellular matrix, migration, focal adhesion, and vascular smooth muscle contraction. The STRING database and Cytoscape tools were used to analyze protein-protein interactions and identify 15 hub candidate genes. Violin plot, boxplot, and prognostic curve analyses were performed using Gene Expression Profiling Interactive Analysis, which identified seven hub genes, including upregulated expressed SPP1 and downregulated expressed MYLK, MYL9, MYH11, CALD1, ACTA2, and CNN1 in PCa compared with normal tissue. Correlation analysis was performed using the OmicStudio tools, which showed that these hub genes were moderately to strongly correlated with each other. Finally, quantitative reverse transcription PCR and western blotting were performed to validate the hub genes, showing that the abnormal expression of the seven hub genes in PCa was consistent with the analysis results of the GEO database.

**Discussion:**

Taken together, MYLK, MYL9, MYH11, CALD1, ACTA2, SPP1, and CNN1 are hub genes significantly associated with PCa occurrence. These genes are abnormally expressed, leading to the formation, proliferation, invasion, and migration of PCa cells and promoting tumor neovascularization. These genes may serve as potential biomarkers and therapeutic targets in patients with PCa.

## 1 Introduction

The human prostate is a walnut-shaped organ located at the base of the bladder. Prostate disease can affect sperm function, which in turn affects male fertility. Prostate cancer (PCa) is a common malignant tumor of the urinary system in middle-aged and elderly men, and ranks second among all malignant tumors worldwide (1). Since patients with early stage PCa generally have no obvious symptoms, most patients are diagnosed at an advanced clinical stage with distant bone metastases, which leads to a high mortality rate. In 2022, the mortality rate of PCa reached 45/10000 deaths in the world and 15.56/100000 deaths in China (2). Therefore, it is important to reveal the potential molecular mechanisms and biomarkers of PCa to provide a basis for the early diagnosis, prevention, and treatment of PCa.

In this study, we used bioinformatics and molecular biology to study the molecular mechanisms of PCa. We downloaded two original microarray datasets GSE55945 and GSE6919 from the National Center for Biotechnology Information (NCBI) Gene Expression Omnibus (GEO) database and searched for differentially expressed genes (DEGs). Gene Ontology (GO) and pathway enrichment analyses were performed to enrich the function of DEGs using the Database for Annotation, Visualization, and Integrated Discovery (DAVID). Protein-protein interaction (PPI) analysis was performed to explore hub candidate genes using the Search Tool for the Retrieval of Interacting Genes/Proteins (STRING) and Cytoscape. Violin plots, boxplots, and prognostic curves were analyzed to identify hub genes using Gene Expression Profiling Interactive Analysis (GEPIA). Finally, hub genes were validated using quantitative reverse transcription PCR (qRT-PCR) and western blotting. This study aimed to provide significant information regarding the molecular mechanisms and biomarkers of PCa.

## 2 Materials and methods

### 2.1 Microarray data extraction

The microarray data used in this study were obtained from the Gene Expression Omnibus (http://www.ncbi.nlm.nih.gov/geo/) database. The gene expression profile data GSE55945 was detected based on the GPL570 [HG-U133_Plus_2] Affymetrix Human Genome U133 Plus 2.0 Array platform, which includes 13 PCa samples and 8 normal prostate samples. The gene expression profile data GSE6919 was analyzed based on the GPL92, GPL93, and GPL8300 platforms, which included 271 PCa samples and 233 normal prostate samples.

### 2.2 Data processing and DEG screening

The microarray data were first preprocessed using the robust multi-array average (RMA), which includes background adjustment, normalization with the quantile method, and expression calculation. Probes were removed when they could not be matched to a specific gene symbol, and the average value was taken as the expression value for each gene when different probes matched to the same gene symbol. Gene expression profiles were analyzed by GEO2R to identify differentially expressed genes in PCa and normal tissues. Finally, genes were annotated using an annotation table downloaded from the GEO website. An absolute value of log2FC (fold change) > 1 and P < 0.05 were used as the screening conditions for DEGs. The distribution of gene expression for GSE55945 and GSE6919 was visualized in the corresponding volcano plot, which visually shows the distribution of DEGs among multiple samples. Venn plot analysis was performed to identify overlapping DEGs that were expressed in GSE55945 and GSE6919.

### 2.3 Functional enrichment analysis

DAVID (https://david.ncifcrf.gov/home.jsp), GO, KEGG (Kyoto Encyclopedia of Gene and Genome, Available online: http://www.genome.jp/kegg), Reactome (Available online: http://www.reactome.org), and BioCyc (Available online: http://biocyc.org) were used to perform GO and signaling pathway enrichment analyses. GO has three independent branches: molecular functions (MF), biological processes (BP), and cellular components (CC). The KEGG, Reactome, and BioCyc databases facilitate the systematic analysis of signaling and intracellular metabolic pathways. Statistical significance was set at P < 0.05 and false discovery rate(FDR) < 0.05.

### 2.4 PPI network analysis

STRING (https://string-db.org/) and Cytoscape (v3.7.2) were used to identify hub candidate genes. ​ STRING is a database that provides a function for predicted protein interactions in which each PPI has one or more ‘scores’ that indicate the confidence in the interaction based on the available evidence. This score ranges from 0 to 1, with 1 representing the highest possible confidence. The interaction relationships between DEGs were determined by PPI network analysis using STRING. Core genes were identified using the cytoHubba plug-in with a confidence PPI score > 0.70 as the threshold. Significant models were obtained from the PPI network based on Molecular Complex Detection (MCODE) analysis in Cytoscape to further filter hub candidate genes. The corresponding genes in the central nodes (node degree > 8) were considered as hub candidate genes with essential physiological regulatory functions. Furthermore, the corresponding pathways in which the hub candidate genes were involved were enriched using Cytoscape.

### 2.5 Determination of hub genes and correlation analysis

Violin plots, boxplots and prognostic curves were executed to evaluate core genes and obtain hub genes using GEPIA (http://gepia.cancer-pku.cn) based on The Cancer Genome Atlas (TCGA) and GTEx databases. Statistical significance was set at P < 0.05. To better understand hub genes, correlation analysis was applied using OmicStudio tools (https://www.omicstudio.cn/tool) and visualized with the “corrplot” R package (v4.2.1).

### 2.6 qRT-PCR

15 Samples were obtained from PCa patients who underwent tumor resection at the Department of Urology, The First Affiliated Hospital of Wenzhou Medical University (Wenzhou, China). Simultaneously, resected 15 samples were obtained from patients with benign prostatic hyperplasia and prostate hypertrophy in the urology department of the hospital as non-cancerous (NC) groups. Detailed clinicopathological data for all enrolled patients were available. The study design was approved by the Ethical Review Board of The First Affiliated Hospital of Wenzhou Medical University, and all patients provided informed consent. All dissected samples were immediately stored in liquid nitrogen until they were prepared for total RNA extraction.

qRT-PCR was performed to confirm the expression of hub genes in the PCa and NC groups. Briefly, total RNA was extracted from the PCa and NC groups using TRIZOL reagent (Life Technologies, Gaithersburg, MD, USA) according to the manufacturer’s instructions. To detect the purity of the RNA, the OD values were measured using a NanoDrop (NanoDrop One, Thermo Fisher Scientific, Waltham, MA, USA). Reverse transcription was performed on each RNA sample (500 μg) using the PrimeScript RT reagent kit with a gDNA eraser (Takara Bio, Dalian, China) according to the manufacturer’s instructions. For qRT-PCR analysis, the reaction mixture (including cDNA, DEPC water, forward primers, and reverse primers) was run in an iQ5 real-time PCR machine (Bio-Rad, Hercules, CA, USA) according to the instructions for SYBR Premix Ex Taq II (Takara Bio, Dalian, China). All the expression levels were normalized to those of the internal control (GAPDH). The qRT-PCR cycles were as follows: step1, preparative denaturation (30 s at 95°C); step 2, 40 cycles of denaturation (5 s at 95°C) and annealing (30 s at 60°C); and step 3, dissociation, following the manufacturer’s protocol. Primer sequences are listed in **Table 1**. The 2^–ΔΔct^ method was used to calculate relative gene expression levels.

**Table 1:** Primer sequences for PCR.

Gene	Forward primer (5’-3’)	Reverse primer (5’-3’)
SPP1	GCCGAGGTGATAGTGTGGTT	AACGGGGATGGCCTTGTATG
MYLK	TGGACGCTGAACGGAAAG	CCTAGCACGGGAGGAAGA
MYL9	AGCCAAGACCACCAAGAAG	TGTCAATGAAGCCATCACG
MYH11	CCGGGAAAACCGAAAACACC	GATGGGCCTTGCGTGATACT
CALD1	AAGAGGGAGGAGATGCGACT	CTCGTCAGGCACACTGTTCT
ACTA2	ACTGCCTTGGTGTGTGACAA	TCCCAGTTGGTGATGATGCC
CNN1	GTTAAGAACAAGCTGGCCCAGAAG	GATGCCATCTTTGAGGCCGT
GAPDH	ACAGTCAGCCGCATCTTCTT	ACGACCAAATCCGTTGACTC

### 2.7 Western blot

The protein expression of specific target hub genes was compared between the PCa and NC groups using western blotting. Total protein was extracted from samples in each group using RIPA Lysis Buffer (50mM Tris•HCl pH 7.6, 150mM NaCl, 1% NP-40, 0.5% sodium deoxycholate, and 0.1% SDS, Boster Biological Technology, Wuhan, China) for 30 min at 4°C before centrifugation at 14,000 rpm for 10 min at 4°C. The total protein concentration was determined using the bicinchoninic acid (BCA, Solarbio Technology) method, and the loading volume was determined based on a protein content of 40 µg/lane. Proteins were separated using sodium dodecyl sulfate–polyacrylamide gel electrophoresis (SDS-PAGE) and transferred to polyvinylidene fluoride (PVDF) membranes. The PVDF membranes were blocked at 37°C for 1.5 h with blocking solution (5% skim milk in Tris-buffered saline Tween (TBST, Boster Biological Technology)), and the membrane was cut according to the molecular weight of the protein of interest based on a pre-stained protein ladder. After washing the membranes twice with TBST, the membranes were incubated with 1:1000 dilutions of primary rabbit monoclonal antibodies against the following proteins or components overnight at 4°C: MYLK, MYL9, MYH11, CALD1, ACTA2, SPP1, and CNN1 (MYLK: ab232949, MYL9: ab64161, MYH11:ab125884, CALD1:ab68878, ACTA2:ab5694, SPP1:ab216406, CNN1:ab78491, Abcam, Cambridge, UK). The membranes were then washed four times with TBST, incubated with goat anti-rabbit-IgG/HRP (1:10,000 dilution; Boster Biological Technology) at 37°C for 1 h, and washed four times with TBST. Images were obtained using an electrochemiluminescence (ECL) method (ECL assay kit, Product Code No.AR1111, Boster Biological Technology). Protein images were digitized using Quantity One software (v4.52) and densitometry was used to quantify protein expression. Data were standardized to the internal control (GAPDH).

## 3 Results

### 3.1 Identification of DEGs

**Figure 1:**
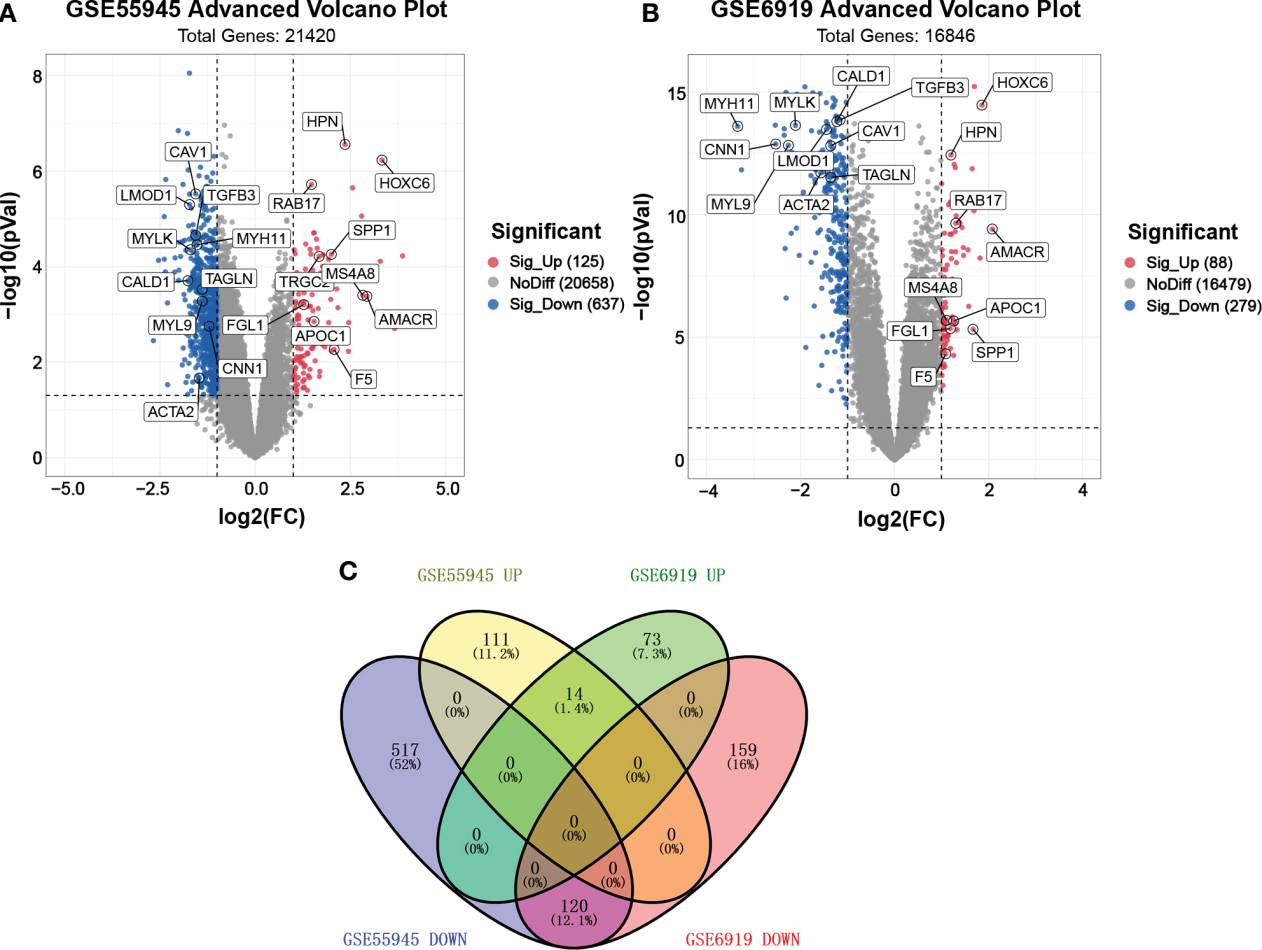
Volcano plot and Venn plot of DEGs. **(A, B)** Volcano plot of DEGs between PCa and normal tissues in GSE55945 and GSE6919, respectively. The X coordinate waslog2 (fold change)and the Y coordinate was −log10 (p value). Each dot represents a gene. Red dots are the upregulated genes of significant expression. Black dots are unchanged expressed genes. **(C)** Venn plot of the overlapping 14 upregulated and 120 downregulated DEGs from the GSE55945 and GSE6919 datasets.

We found two samples, GSE55945 and GSE6919, by searching GEO DataSets. ​ A total of 762 DEGs were identified through the gene expression profile data GSE55945, containing 13 PCa samples and 8 normal prostate samples (**Figure 1A**). A total of 367 DEGs were obtained from the gene expression profile data GSE6919, containing 271 PCa samples and 233 normal prostate samples (**Figure 1B**). A Venn plot was used to identify the DEGs appearing in both databases. A total of 134 common DEGs were screened, including 14 upregulated and 120 downregulated genes (**Figure 1C**).

### 3.2 GO enrichment analysis

**Figure 2:**
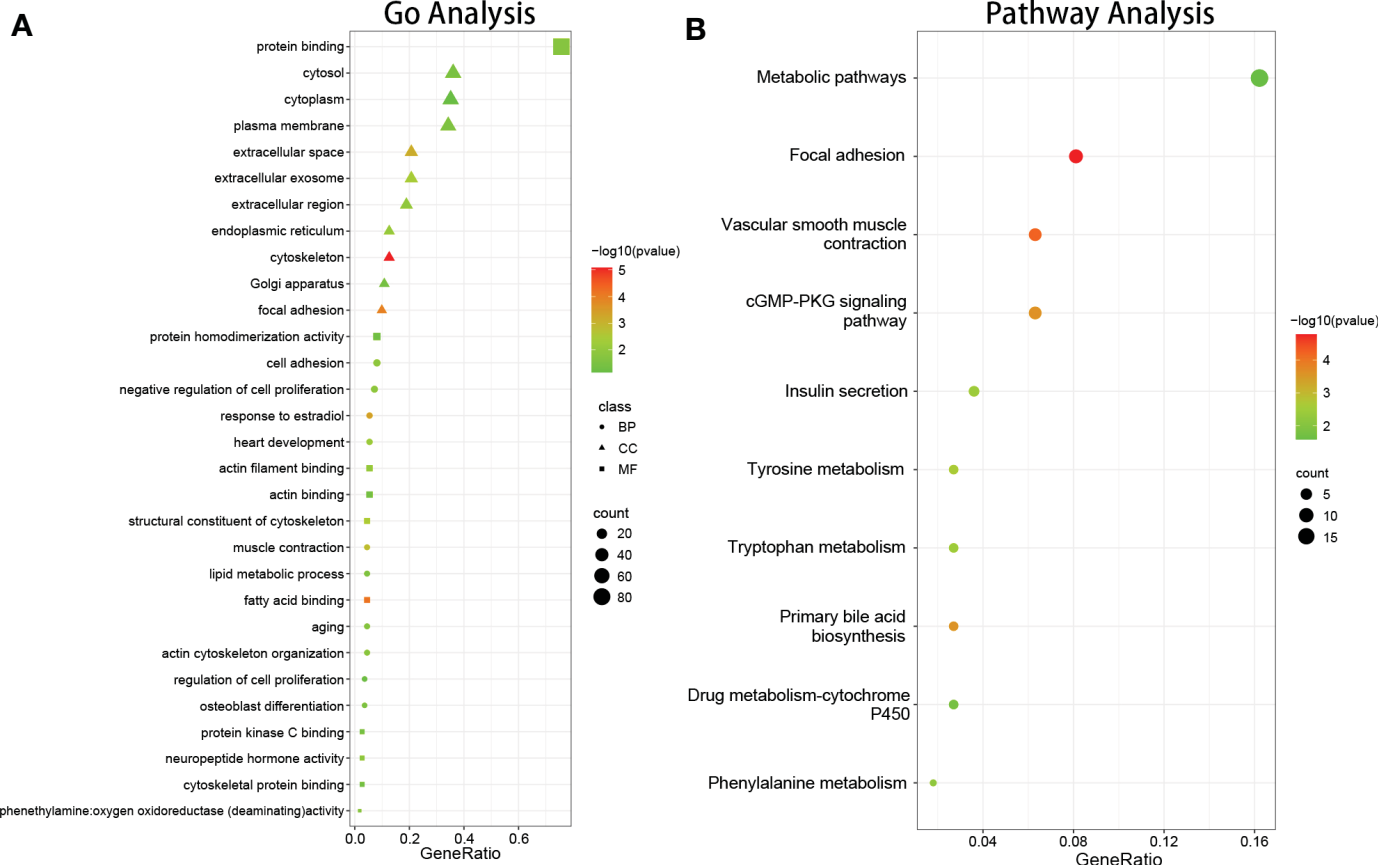
The results of function and pathway enrichment analysis in DEGs. **(A)** The bubble plots indicate the results of function enrichment analysis in DEGs, including biological process, cell component, and molecular function. **(B)** The bubble plots indicate the results of pathway enrichment analysis in DEGs.

GO enrichment analysis of DEGs was conducted using DAVID, based on the GO database. GO terms with P < 0.05 (FDR<0.05) and contained at least two genes were selected. As shown in **Figure 2A**, CC analysis indicated that DEGs were most enriched in the cytosol, plasma membrane, extracellular space, extracellular exosome, extracellular region, cytoskeleton, endoplasmic reticulum, Golgi apparatus, focal adhesion, and extracellular matrix. For BP, the most significantly altered pathways were cell adhesion, negative regulation of cell proliferation, response to estradiol, heart development, muscle contraction, actin cytoskeleton organization, aging, lipid metabolic process, intermediate filament organization, and regulation of cell migration. For MF, protein binding, actin filament binding, actin binding, fatty acid binding, structural constituent of cytoskeleton, neuropeptide hormone activity, protein kinase C binding, cytoskeletal protein binding, aliphatic-amine oxidase activity, and phenethylamine:oxygen oxidoreductase (deaminating) activity were most significantly enriched. The results showed that most of the genes involved in the aforementioned GO terms were downregulated.

### 3.3 Signaling pathway enrichment analysis

DEG signaling pathway enrichment was conducted using DAVID based on the online databases KEGG, Reactomen, and BioCyc. Signaling pathway analysis showed that the DEGs were involved in vascular smooth muscle contraction, focal adhesion, tyrosine metabolism, phenylalanine metabolism, and the primary bile acid biosynthesis pathway (**Figure 2B**).

### 3.4 PPI network construction

**Figure 3:**
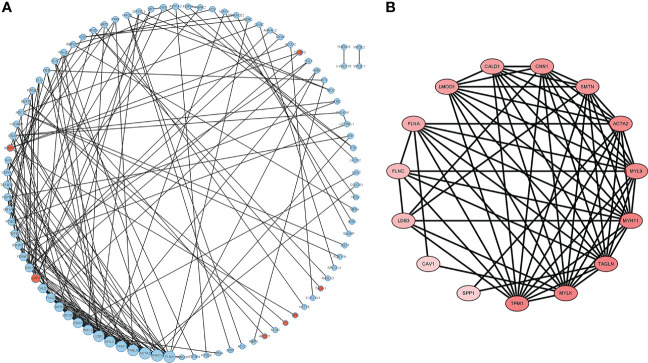
PPI network complex and modular analysis of DEGs in PCa. The network nodes are proteins. The edges represent the predicted functional associations. **(A)** Using the STRING Website, a total of 134 DEGs (red dots represent upregulated genes and blue dots represent downregulated genes) were filtered into the DEGs PPI network complex. The node size reflects the density of the nodes and the surrounding nodes. **(B)** The most significant module was screened using MCODE in the Cytoscape software. The higher degree of DEGs is represented by a more intense red color.

The DEGs were filtered into the DEGs PPI network complex using the STRING database. In this study, 134 simultaneous DEGs were uploaded to the STRING website for PPI analysis. The PPI network results are shown in **Figure 3**. Ninety-three core genes were obtained by selecting a PPI score > 0.7 (**Figure 3A**). Fifteen hub candidate genes were further identified by filtering the node degree > 8 criteria, including ACTA2, FLNA, MYH11, TAGLN, LDB3, MYLK, TPM1, MYL9, CNN1, FLNC, LMOD1, SMTN, CALD1, SPP1, and CAV1 (**Figure 3B**). These genes mainly participate in the focal adhesion and vascular smooth muscle contraction pathways.

### 3.5 Determination of hub genes and correlation analysis

**Figure 4:**
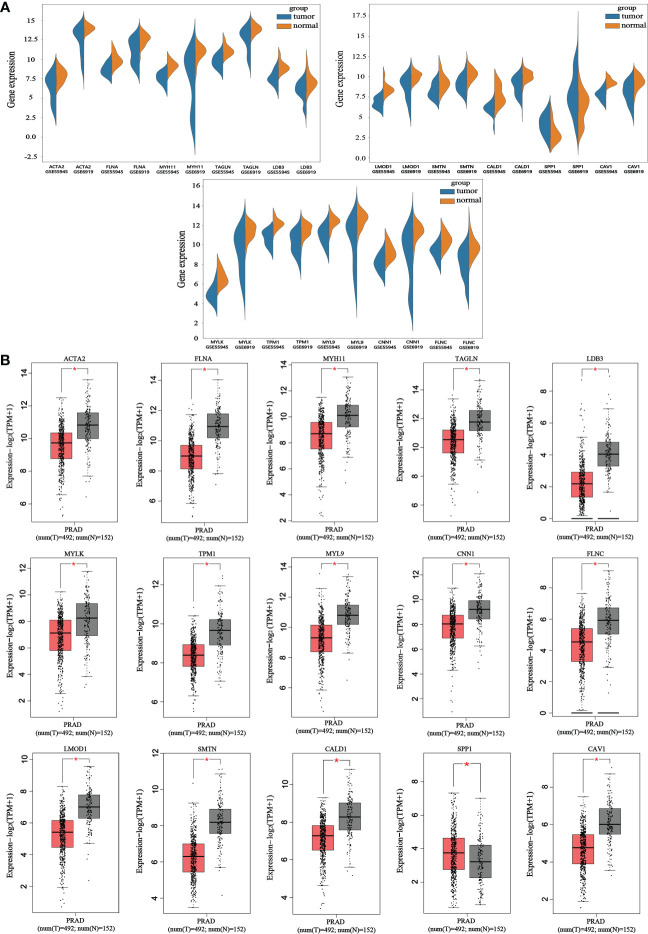
Violin plots and boxplots of 15 hub candidate genes using GEPIA. **(A)** Violin plots were analyzed to compare the expression distribution of 15 hub candidate genes in the GSE6919 and GSE55945 datasets. **(B)** Boxplots were performed to validate the expression of 15 hub candidate genes in PCa samples compared with normal samples based on TCGA and GTEx dataset. ACTA2, FLNA, MYH11, TAGLN, LDB3, MYLK, TPM1, MYL9, CNN1, FLNC, LMOD1, SMTN, CALD1, and CAV1 genes had significantly downregulated expression whereas SPP1 had significantly upregulated expression in PCa compared to normal specimen (*P < 0.05 versus normal group). The red and gray boxes represent PCa and normal tissues, respectively. The dots represent the expression in each sample.

**Figure 5:**
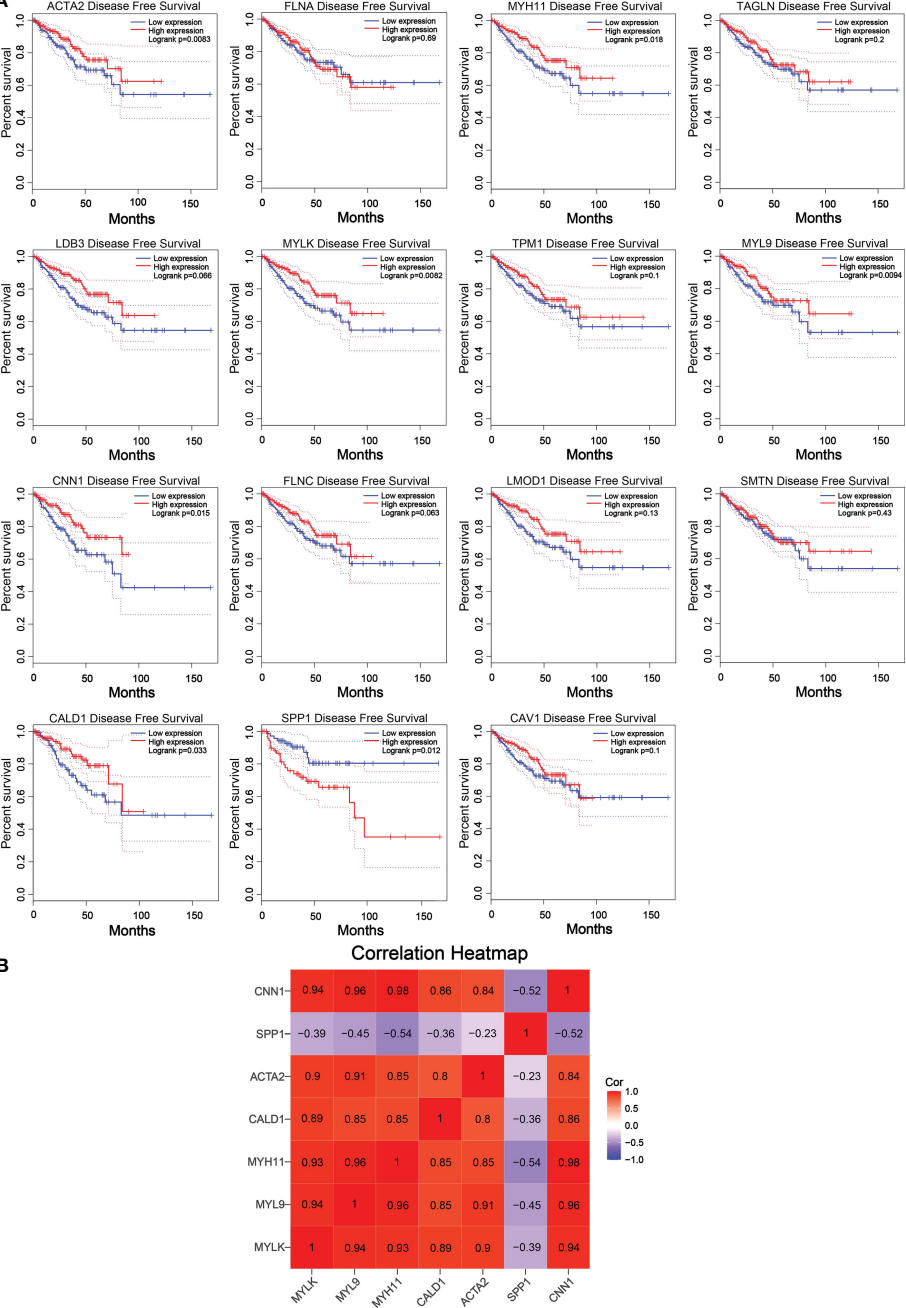
Prognostic curves of 15 hub candidate genes using GEPIA and a correlation matrix of seven hub genes proportions. **(A)** Prognostic curves were analyzed based on the TCGA and GTEx datasets. Downregulation of the genes MYLK, MYL9, MYH11, CALD1, ACTA2, and CNN1 and upregulation of SPP1 showed a significantly worse survival rate (P < 0.05) compared to that of the control, while the prognosis curves of the other eight genes were without statistical significance. The red lines represent patients with high gene expression, and blue lines represent patients with a low gene expression. **(B)** Correlation analysis performed using OmicStudio tools. Red represents positive correlations, and blue represents negative correlations.

The reliability of the hub genes in the two datasets was confirmed. The violin plot revealed the expression of 15 hub candidate genes that were highly consistent in the GSE6919 and GSE55945 datasets (**Figure 4A**). Boxplots and prognostic curves were analyzed using GEPIA based on TCGA and GTEx datasets (**Figures 4B**, **5A**). The boxplot results indicated that all the downregulated genes identified in this study, including ACTA2, FLNA, MYH11, TAGLN, LDB3, MYLK, TPM1, MYL9, CNN1, FLNC, LMOD1, SMTN, CALD1, and CAV1, were also significantly downregulated in TCGA prostate cancers, and the upregulated gene SPP1 was also found to be significantly overexpressed in TCGA prostate cancers. It was found that the downregulation of MYLK, MYL9, MYH11, CALD1, ACTA2, and CNN1, and the upregulated expression of SPP1, were correlated with significantly worse overall survival in PCa patients, while FLNA, TAGLN, LDB3, TPM1, FLNC, LMOD1, SMTN, and CAV1 expression was not associated with survival (P > 0.05). Hub genes were screened and identified according to the violin plot, boxplots, and prognostic curves results, including MYLK, MYL9, MYH11, CALD1, ACTA2, SPP1, and CNN1. Further analysis revealed that these hub genes were moderately to strongly correlated with each other. The results of the correlation analysis of these genes are shown in **Figure 5B**.

### 3.6 Validation of hub genes

**Figure 6:**
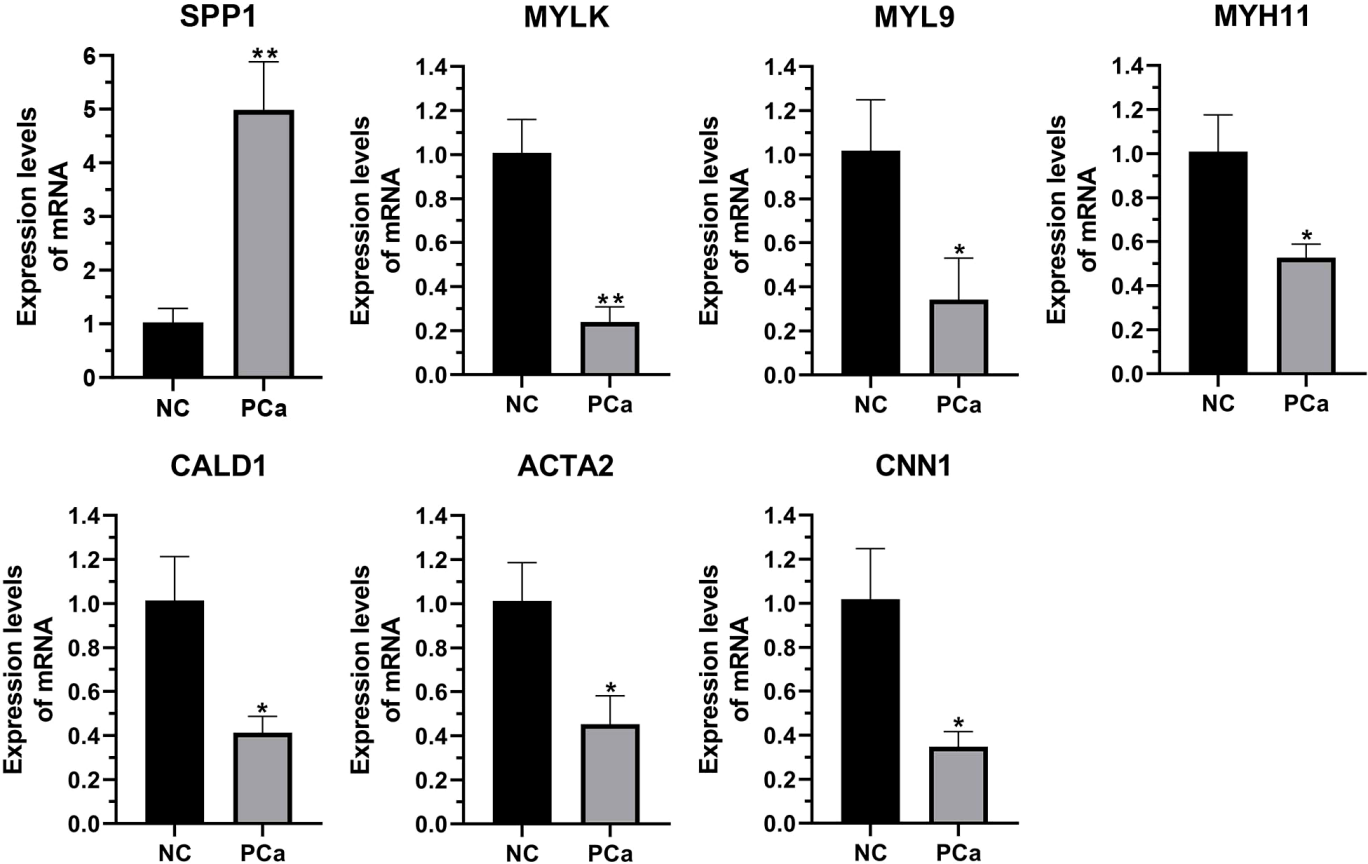
The transcription of seven hub genes (MYLK, MYL9, MYH11, CALD1, ACTA2, SPP1, and CNN1) in the PCa and non-cancerous (NC) groups, as detected by qRT-PCR. *p< 0.05; **p< 0.01 versus NC group. The expression of the seven hub genes was normalized to the internal control (GAPDH) using the 2^–ΔΔct^ method.

**Figure 7:**
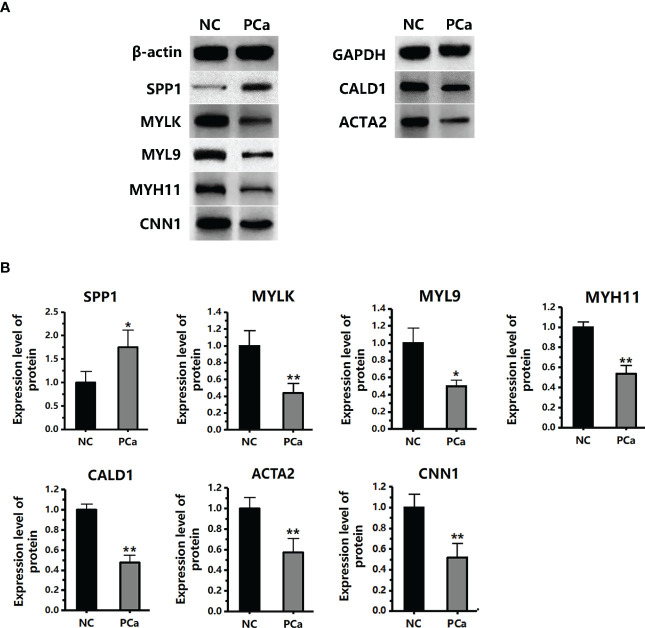
The protein expression level of seven hub genes (MYLK, MYL9, MYH11, CALD1, ACTA2, SPP1, and CNN1) in the PCa and non-cancerous (NC) groups, as detected by western blotting. **(A)** The protein images were obtained using ECL. **(B)** The protein image was digitized using Quantity One software and densitometry was used to quantify protein expression. *P< 0.05; **P< 0.01 versus NC group. The expression of the seven proteins was normalized to the internal control (β-actin and GAPDH).

We performed qRT-PCR on clinical samples to further verify the expression of hub genes. Compared to the NC group, the PCa group showed significantly increased mRNA levels of SPP1 (P < 0.05) and significantly decreased mRNA levels of MYLK, MYL9, MYH11, CALD1, ACTA2, and CNN1 (P < 0.05) (**Figure 6**). Next, western blotting was performed to assess the hub protein content. SPP1 protein levels were significantly upregulated in the PCa group compared to those in the NC group, whereas those of MYLK, MYL9, MYH11, CALD1, ACTA2, and CNN1 were significantly downregulated (**Figure 7**). These results were consistent with the findings of the bioinformatics analysis.

## 4 Discussion

This study integrated two profile datasets from different groups, utilized bioinformatics methods to deeply analyze these datasets, and identified 134 commonly altered DEGs (14 upregulated and 120 downregulated) in the first step. Next, the DEGs were subjected to GO enrichment analysis, which showed that these DEGs were significantly enriched in the functional modules, cellular components, and biological processes of cancer development. Some of the significant characteristics of PCa include cell adhesion, extracellular matrix (ECM), and cell migration.

Cell adhesion is the process by which similar cells aggregate to form clusters of cells or tissues and play a role in the development of inflammation, tumor metastasis, and the healing of tissue damage (3). Cell adhesion is a key mediator of cancer progression. It is also a marker for cancer metastasis. Tumor cell migration is acquired during the continuous process of adhesion contact and adhesion release. First, the matrix metalloproteinases (MMPs) secreted by tumor cells degrade the ECM of tumor cells, resulting in adhesion molecule deficiency and reduced adhesion between tumor cells (4). The adhesion between tumor cells and normal tissues is enhanced by laminin (LN), a component of the basement membrane (BME), combined with integrin receptors on the surface of tumor cells. Furthermore, tumor cells degrade the ECM between tumor cells and normal tissues by releasing MMPs and invading normal cells through natural voids in the basement membrane or forcibly through the voids. Tumor cells begin to proliferate uncontrollably, invading and crowding out the surrounding normal tissues to interfere with or disrupt their function. During PCa development, malignant luminal epithelial cells change from tumor cell-cell adhesion to cell-ECM adhesion, which leads to weakened adhesion between tumor cells and causes tumor metastasis and invasion (5). In our study, genes associated with adhesion function were enriched, including SPP1, CLDN8, COL4A6, RND3, DPT, EFS, TNC, AOC3, and TGFB1I1. SPP1 and CLDN8 were upregulated in prostate tumor tissues, whereas COL4A6, RND3, DPT, EFS, and TGFB1I1 were downregulated. SPP1 is highly expressed in PCa tissues and the binding of SPP1 to receptors on the cell surface can promote cell movement and adhesion enhancement (6). It can also activate the PI3K/AKT and ERK1/2 signaling pathways and promote the proliferation and invasion of PCa cells (6). CLDN8 regulates intracellular signal transduction and stabilizes the cytoskeleton, and its upregulation promotes the proliferation and migration of PCa cells (7). RND3 is a tumor suppressor gene in PCa. Overexpression of RND3 in PCa cells can lead to cell growth arrest and death; conversely, its low expression can lead to ECM adhesion plaque loss, which increases the metastatic potential of tumor cells (8). COL4A6 is significantly downregulated in metastatic PCa tissues, promoting cell migration and invasion (9). DPT is an ECM protein that plays a role in localized adherence, and its downregulation accelerates PCa cell proliferation and tumor metastasis (10). The expression of EFS is downregulated in PCa, and the low-level expression of EFS promotes the proliferation, migration, and colony formation of PCa cells (11). Upregulation of TNC can increase tumor cell metastasis to lymph nodes and bones *in vivo* (12). AOC3 is an endothelial adhesion protein that is involved in tumor cell extravasation and can mediate tumor infiltration into lymphocytes and invasion into the endothelial cell layer of other organs and tissues (13). TGFB1I1, also known as Hic-5/ARA55, is significantly reduced in PCa tissues and promotes the deterioration of PCa by promoting the epithelial-to-mesenchymal transition induced by TGF-β (14). Therefore, our study showed that abnormal expression of SPP1, CLDN8, COL4A6, RND3, DPT, EFS, TNC, AOC3, and TGFB1I1 during the development of PCa could mediate abnormal changes in the adhesion properties of PCa tissues, thus promoting the metastasis and invasion of tumor cells.

As a scaffold for tissues, ECM provides key biochemical and biomechanical clues that guide cell growth, survival, migration, differentiation, and regulation of vascular development and immunity. The ECM is a significant factor in regulating tumor metastasis and invasion. Abnormal changes in the ECM between tumor cell-tumor cell and tumor cell-normal cells can promote tumor metastasis and invasion into normal tissues. In addition, ECM can regulate tumor microenvironment angiogenesis, which can provide nutrients for tumor growth and promote tumor proliferation and migration (15). Studies have found that the alteration of the ECM is related to the differentiation and development of PCa, in which the incompleteness or loss of the basement membrane provides conditions for the metastasis of PCa cells (16). In well-differentiated PCa, the ECM is distributed continuously and linearly, and the microvessel density (MVD) is low. However, in poorly differentiated PCa, the ECM is fragmented or disintegrated, and the MVD is significantly higher than that of tumor tissue without metastasis. Genes related to ECM function enriched in our study included CRISP3, OGN, SPOCK3, COL4A6, CCBE1, and FLRT3. CRISP3 was upregulated; conversely, OGN, SPOCK3, COL4A6, CCBE1, and FLRT3 were downregulated in prostate tumor tissues. CRISP3 is an ECM protein found in the male reproductive tract and its high expression enhances the motility and aggressiveness of PCa cells. CRISP3 promotes the progression of PCa from carcinoma *in situ* to invasive carcinoma via changes in cell adhesion pathways. The higher its expression, the higher the risk of relapse in patients (17). OGN is involved in the formation and regulation of ECM, and low expression of OGN is involved in the regulation of tumor lymphatic metastasis, promotion of angiogenesis, and promotion of invasion and metastasis of tumor cells (18). SPOCKs are a class of ECM proteoglycans whose low expression can increase tumor aggressiveness (19). CCBE1 plays a role in regulating ECM remodeling and migration. Deletion of CCBE1 synergizes with vascular endothelial growth factor to promote tumor cell migration and is associated with reduced overall survival of patients (20). Moreover, it has been found to CCBE1 promotes angiogenesis in colorectal cancer (21). FLRT3 regulates cell-cell adhesion. The absence of FLRT3 causes changes in cell polarity, decreased adhesion, and enhanced cell motility, thus playing a role in tumor metastasis and invasion. In addition, it mediates the signal transduction of vascular endothelial growth factor, which plays a role in the generation of blood vessels (22). Therefore, our study found that during PCa pathogenesis, the disorganized expression of CRISP3, OGN, SPOCK3, COL4A6, CCBE1, and FLRT3 leads to ECM dysfunction and promotes angiogenesis and tumor development.

Cell migration is a form of cell movement generated by cells receiving migration signals and involves fundamental physiological processes such as embryonic development, tissue homeostasis, immune surveillance, and wound healing. In cancer tissues, cells, driven by chemokine concentration gradients, clear the barriers of ECM and BME and enter adjacent or systemic tissues to complete migration and diffusion, leading to the accelerated death of patients (23). In PCa, PCa cells first invade the surrounding tissues or adjacent organs in the early stage and metastasize to the bone through the blood in the late stage. The enriched genes related to cell migration in our study included FLNA, DPYSL3, KRT5, and TNC, all of which were downregulated in PCa tissues. Low expression of FLNA promotes the loss of function of MMPs, which is closely related to cell adhesion, proliferation, migration, signal transduction, and tumorigenesis (24). DPYSL3 is a cell adhesion molecule, and its low expression in PCa cells significantly promotes cancer cell migration and invasion; its concentration is directly proportional to the prognosis of patients (25). The KRT5 gene can regulate the formation of the cytoskeleton and venous invasion of cancer cells, and its low expression is related to tumor recurrence and metastasis (26). As a member of the ECM, TNC is abnormally expressed in the process of prostate tumorigenesis, which can promote an increase in microvessel density and is related to proliferation, angiogenesis, adhesion, invasion, and other processes of PCa cells (27). Therefore, our study revealed that low expression of FLNA, DPYSL3, KRT5, and TNC promotes tumor migration and development during the pathogenesis of PCa.

GO analysis showed that abnormal cell adhesion, ECM, and cell migration promoted PCa progression. The same results were verified by KEGG pathway enrichment analysis. The focal adhesion and vascular smooth muscle contraction pathways were shown in the KEGG enrichment analysis. The focal adhesion pathway is involved in cell adhesion, and irregularity leads to tumor migration and invasion. The vascular smooth muscle contraction pathway is involved in vascular smooth muscle contraction and relaxation. Excessive constriction of blood vessels leads to vascular hypoxia, which promotes the formation of new blood vessels and provides growth conditions for cancer cells (28). Excessive constriction of blood vessels leads to high blood pressure, which causes the blood vessels to lose oxygen. Angiogenic factors such as vascular endothelial growth factor (VEGF) and angiotensin II (Ang-II) are abnormally produced in hypoxic environment (29). VEGF binds to VEGF receptors on the endothelial cell membrane, thereby activating mitogen-activated protein kinase (MAPK) and inducing endothelial cell proliferation (30). Ang-II can lead to disconnection between endothelial cells and surrounding cells, resulting in vascular instability and endothelial activation (31). VEGF cooperates with Ang-II to induce endothelial cells to receive the budding signal of VEGF and other angiogenesis inducers (32). Neovascularization occurs continuously in tumor tissues and promotes the development of tumor cells. One study showed that during PCa progression, new blood vessels are necessary to promote tumor cell survival and proliferation (33). Our results on the enrichment pathway suggest that, in the above KEGG pathway analysis results, abnormalities in the focal adhesion and vascular smooth muscle contraction pathways will lead to changes in adhesion properties and abnormal vasoconstriction, which will lead to the migration of tumor cells and generation of new blood vessels in tumor tissue.

ACTA2, FLNA, MYH11, TAGLN, LDB3, MYLK, TPM1, MYL9, CNN1, FLNC, LMOD1, SMTN, CALD1, SPP1, and CAV1 were obtained from 134 DEGs through the PPI network and node degree. Among these genes, SPP1 expression was upregulated, whereas the others were downregulated. These genes were subjected to violin plot, boxplot, and prognostic curve analyses. The violin plot showed that the expression of 15 hub candidate genes was consistent in the GSE6919 and GSE55945 datasets. Prognostic curves showed that the downregulation of MYLK, MYL9, MYH11, CALD1, ACTA2, and CNN1, and the high expression of SPP1 were correlated with significantly worse overall survival in PCa patients, while FLNA, TAGLN, LDB3, TPM1, FLNC, LMOD1, SMTN, and CAV1 expression was not associated with survival (P > 0.05). The boxplot results showed that all upregulated and downregulated genes had the same expression tendency in TCGA prostate cancers. In addition, qRT-PCR and western blotting indicated that compared with the NC group, the mRNA and protein levels of MYLK, MYL9, MYH11, CALD1, ACTA2, SPP1, and CNN1 were differentially expressed in the PCa group, which is consistent with the analysis results of GEO datasets. Therefore, MYLK, MYL9, MYH11, CALD1, ACTA2, SPP1, and CNN1 might be hub genes that play a significant role in the development of PCa.

Among these, myosin light chain kinase (MYLK) and myosin light chain 9 (MYL9) participate in cell adhesion and smooth muscle contraction. MYLK is a calcium ion (Ca2+)/calmodulin (CaM)-dependent enzyme that is ubiquitously expressed in various tissues, including smooth muscle and nonmuscle cells. MYLK is composed of actin-binding, kinase, and myosin-binding domains. It has been shown that MYLK principally contributes to a variety of biological process that are related with myosin activation such as cell adhesion, migration, division, invasion, and constriction (34). Upregulated MYLK combined with Rho-associated kinase (ROCK) catalyzes the phosphorylation of MYL9, which is a key step in cell adhesion and smooth muscle cell contraction (35). MYL9 is an actin‐dependent molecular motor that uses the energy of ATP hydrolysis to move along actin filaments and generate force (36). MYL9 regulates actin alpha 2 (ACTA2) polymerization to form pseudopods that regulate cell adhesion and migration (37). Studies have shown that upregulation of MYL9 and ACTA2 promotes migration in most cancers, but their downregulation promotes migration in a few cancers. For instance, low expression of MYL9 was detected in clinical hepatocellular carcinoma specimens, which improved the migration and invasion of hepatocellular carcinoma cells (38). In PCa patients, low MYL9 expression is positively associated with poor prognosis (39). Our results showed that MYLK, MYL9, and ACTA2 were downregulated in PCa, which may be key factors in promoting metastasis and invasion of PCa cells. On the other hand, MYLK and phosphorylated MYL9 can also directly affect the interaction between ACTA2 and myosin, thereby regulating smooth muscle (SM) activity. MYL9 binds to ACTA2 via myosin heavy chains (MHC/MYH) to form a crossbridge that participates in SM contraction (40). Vasoconstriction is an important factor that promotes the formation of new blood vessels and occurrence of tumors (41). Our results showed that MYLK, MYL9, ACTA2, and MYH11 are downregulated in PCa, which may promote abnormal SM contraction, resulting in the formation of new blood vessels and promoting the occurrence and development of PCa. Besides, ACTA2 is positively controlled by Caldesmon (CALD1) in the process of SM contraction. CALD1 is an actin-myosin-binding protein found on thin filaments of SM and nonmuscle cells. Phosphorylated CALD1 binding to ACTA2 promotes actin and myosin binding and smooth muscle contraction, which are important processes in the regulation of SM cell contraction (42). Our results show that CALD1 is downregulated in PCa, which promotes abnormal vasoconstriction and PCa development.

Secreted phospho-protein-1 (SPP1), also known as osteopontin (OPN), is an ECM protein and its abnormal expression is closely related to tumors. SPP1 acts as a regulator of cell-ECM interactions and regulates the downstream pathways of migration, adhesion, division, proliferation, and survival. High expression of SPP1 leads to the enhancement of cell migration and adhesion, acceleration of cell growth and division, and abnormal prolongation of cell survival time, which promotes the proliferation, migration, differentiation, and immune escape of tumor cells (43). SPP1 is highly expressed in cervical cancer tissues and downregulation of SPP1 induces apoptosis in cervical cancer cells (44). SPP1 expression is upregulated in lung adenocarcinoma and colon cancer and its upregulation is associated with decreased patient survival and cancer metastasis (45). Our results showed that it is also upregulated in PCa, possibly through adhesion, proliferation, migration, and differentiation pathways, leading to the development of PCa.

Calponin 1 (CNN1) is a regulator of actomyosin contraction and arrests the G2 to M phase transition during cell proliferation. It has been documented that CNN1 can inhibit tumor cell formation, proliferation, and colony formation as a tumor suppressor (46). It has been reported that CNN1 is lowly expressed in bladder cancer, liver cancer, and lung squamous cell carcinoma, which were associated with decreased overall survival (47). CNN1 knockdown increases the growth rate, volume, and weight of tumors while accelerating proliferation and angiogenesis in colorectal cancer (48). Based on our study, we found that the expression of CNN1 in PCa was downregulated, indicating that the downregulation of CNN1 may promote the formation and proliferation of PCa cells. Therefore, CNN1 may also serve as a biomarker for PCa development.

In summary, MYLK, MYL9, MYH11, CALD1, ACTA2, SPP1, and CNN1 may be hub genes involved in the occurrence and development of PCa. Abnormal expression of these genes can promote the formation, proliferation, migration, and invasion of PCa cells, as well as tumor angiogenesis. These findings could significantly improve our understanding of the causes and underlying molecular events in PCa, and these hub genes could be therapeutic targets for PCa.

## 5 Conclusions

This study focused on using bioinformatics methods to analyze gene expression in PCa tissues, and 134 PCa DEGs were identified. GO and KEGG enrichment analyses showed that these DEGs were involved in biological processes such as cell adhesion, ECM, migration, focal adhesion, and vascular smooth muscle contraction. Among the DEGs, seven hub genes, MYLK, MYL9, MYH11, CALD1, ACTA2, SPP1, and CNN1, were acquired. Their abnormal expression leads to the formation, proliferation, invasion, and migration of PCa cells, and promotes tumor neovascularization. These seven hub genes may serve as potential biomarkers and therapeutic targets for patients with PCa. In the future, more studies need to be conducted to support the results we found and to identify additional molecular mechanisms of PCa.

## Data availability statement

The original contributions presented in the study are included in the article/supplementary material. Further inquiries can be directed to the corresponding author.

## Ethics statement

The studies involving human participants were reviewed and approved by Ethics Committee in Clinical Research of the First Affiliated Hospital of Wenzhou Medical University, Wenzhou Medical University. The patients/participants provided their written informed consent to participate in this study.

## Author contributions

XH developed the idea and obtained the fund. HZ and XH designed the study, executed the experiments, analyzed the data, and drafted and wrote the manuscript. QL provided support and assistance in the writing of the manuscript. XG provided experimental resources and critically evaluated the manuscript.

## Glossary

**Table:**

Ang-II	Angiotensin II
ACTA2	Actin alpha 2
BP	Biological process
BME	Basement Membrane
CC	Cellular component
Ca2+	Calcium ion
CaM	Calmodulin
CALD1	Caldesmon
CNN1	Calponin 1
DEGs	Differentially expressed genes
DAVID	Database for Annotation aVisualization and Integrated Discovery
ECL	Electrochemiluminescence
ECM	Extracellular matrix
GEO	Gene Expression Omnibus
GO	Gene Ontology
GEPIA	Gene Expression Profiling Interactive Analysis
KEGG	Kyoto Encyclopedia of Gene and Genome
log2FC	Log2 fold change
LN	Laminin
MF	Molecular function
MCODE	Molecular Complex Detection
MMPs	Matrix Metalloproteinases
MVD	Microvessel density
MAPK	Mitogen-activated protein kinase
MYLK	Myosin light chain kinase
MYL9	Myosin light chains 9
MHC/MYH	Myosin heavy chains
NCBI	National Center for Biotechnology Information
NC	Non-cancerous
OPN	Osteopontin
PCa	Prostate cancer
PPI	Protein-protein interaction network
PVDF	Polyvinylidene fuoride
qRT-PCR	Quantitative reverse transcription PCR
RMA	Robust multi-array average
ROCK	Rho-associated kinase
STRING	Search Tool for the Retrieval of Interacting Genes/Proteins
SDS-PAGE	Sodium dodecyl sulfate&ndash;polyacrylamide gel electrophoresis
SM	Smooth muscle
SPP1	Secreted phospho-protein-1
TCGA	The Cancer Genome Atlas
TBST	Tris-buffered saline Tween
VEGF	Vascular endothelial growth factor
